# REgistry Of Catheter AbLation After Congenital Heart Disease Surgery(REAL‐CHD Registry)

**DOI:** 10.1002/joa3.70347

**Published:** 2026-04-20

**Authors:** Hitoshi Mori, Nobuhiro Nishii, Yoshiaki Kato, Takahiko Kinjo, Tsugutoshi Suzuki, Jun Yoshimoto, Shigeru Tateno, Aya Miyazaki, Hisaaki Aoki, Satoshi Higuchi, Koji Nakagawa, Keiko Toyohara, Daiji Takeuchi, Tetsuri Sakai, Kiyotaka Takefuta, Ryuta Henmi, Masayoshi Mori, Daigo Yagishita, Shun Hasegawa, Yuko Matsui, Shonosuke Watanabe, Kensuke Kikuchi, Kunihiro Kani, Yasunori Hiranuma, Toshiya Matsuyama, Kazuhisa Matsumoto, Masataka Narita, Wataru Sasaki, Taisuke Nabeshima, Kota Nagaoka, Takuro Kojima, Ritsushi Kato, Morio Shoda, Naokata Sumitomo

**Affiliations:** ^1^ Department of Pediatric Cardiology Saitama Medical University International Medical Center Saitama Japan; ^2^ Department of Cardiology Saitama Medical University International Medical Center Saitama Japan; ^3^ Department of Cardiovascular Therapeutics Okayama University Graduate School of Medicine, Dentistry, and Pharmaceutical Sciences Okayama Japan; ^4^ Department of Pediatric Cardiology National Cerebral and Cardiovascular Center Osaka Japan; ^5^ Department of Pediatric and Adult Congenital Cardiology Tokyo Women's Medical University Tokyo Japan; ^6^ Department of Pediatric Electrophysiology Osaka City General Hospital Osaka Japan; ^7^ Department of Electrophysiology Shizuoka Children's Hospital Shizuoka Japan; ^8^ Department of Pediatrics and Adult Congenital Heart Disease Chiba Kaihin Municipal Hospital Chiba Japan; ^9^ Department of Pediatric Cardiology, Department of Adult Congenital Heart Disease Seirei Hamamatsu General Hospital Shizuoka Japan; ^10^ Department of Cardiology Osaka Women's and Children's Hospital Osaka Japan; ^11^ Clinical Research Division for Heart Rhythm Management, Department of Cardiology Tokyo Women's Medical University Tokyo Japan; ^12^ Department of Cardiovascular Medicine Okayama University Graduate School of Medicine, Dentistry, and Pharmaceutical Sciences Okayama Japan; ^13^ Department of Cardiology Seirei Hamamatsu General Hospital Shizuoka Japan; ^14^ Department of Cardiology Tokyo Women's Medical University Tokyo Japan; ^15^ Department of Cardiology Chiba Cerebral and Cardiovascular Center Chiba Japan

**Keywords:** artificial patch, catheter ablation, congenital heart disease, prosthetic valve

## Abstract

**Background:**

Advances in surgical management have improved long‐term survival in patients with congenital heart disease (CHD), leading to a growing population of adults with postoperative arrhythmias. However, contemporary data on catheter ablation practice and outcomes in patients with CHD remain limited.

**Methods:**

This multicenter, retrospective registry study included patients who underwent catheter ablation for postoperative CHD‐related arrhythmias between April 2007 and December 2025 at 10 centers. CHD severity was classified as mild, moderate, or severe according to Japanese Circulation Society guidelines. Procedural characteristics, arrhythmia profiles, acute procedural outcomes, and complications were analyzed. Associations between age, disease severity, arrhythmia burden, and procedural success were evaluated using regression analyses.

**Results:**

A total of 1 719 patients were included. The number of ablation procedures increased over time across all CHD severity categories. Increasing age was associated with lower disease severity, whereas patients with severe CHD, particularly those after a Fontan repair, underwent ablation at younger ages. Procedural success decreased with increasing CHD severity (complete success: 92.5% in mild, 81.4% in moderate, and 71.4% in severe CHD) and with a greater number of induced arrhythmia types. Overall, procedure‐related complications occurred in 2.3% of patients, with worsening heart failure being the most frequent non‐vascular adverse event.

**Conclusion:**

In a large contemporary Japanese cohort, catheter ablation for postoperative CHD‐related arrhythmias was increasingly performed and demonstrated acceptable safety. However, procedural success was lower in patients with more severe CHD and greater arrhythmia complexity, highlighting the need for specialized ablation strategies and careful periprocedural management in this growing population.

## Graphical abstract

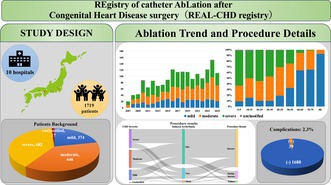

**Abbreviations**
AF: atrial fibrillation
APC: atriopulmonary connection
ASD: atrial septal defect
AT: atrial tachycardia
AVB: atrioventricular block
AVNRT: atrioventricular nodal reentrant tachycardia
AVP: aortic valve plasty
AVR: aortic valve replacement
AVRT: atrioventricular reentrant tachycardia
CHD: congenital heart disease
JET: junctional ectopic tachycardia
LA: left atrium
LT: lateral tunnel
LV: left ventricle
MVP: mitral valve plasty
MVR: mitral valve replacement
PAC: premature atrial contraction
PV: pulmonary vein
PVC: premature ventricular contraction
RA: right atrium
RV: right ventricle
SVC: superior vena cava
TIA: transient ischemic attack
VT: ventricular tachycardia

## 1 Introduction

Advances in surgical techniques and perioperative management have substantially improved the long‐term survival of patients with congenital heart disease (CHD) over the past half‐century [1, 2]. As a result, the population of adults living with repaired CHD has steadily increased [3]. However, improved survival has also led to the emergence of new clinical challenges in this growing cohort.

Surgical scars, altered cardiac anatomy, and chronic hemodynamic stress contribute to the development of arrhythmogenic substrates, and the prevalence of arrhythmias has been shown to increase with advancing age in patients with CHD [4, 5, 6, 7]. These arrhythmias represent one of the most common causes of unplanned hospital admissions in the late postoperative period, and emergency hospitalizations due to arrhythmias have been associated with an unfavorable prognosis [8].

Although optimal arrhythmia management is therefore crucial in this population, therapeutic options remain limited [5]. Antiarrhythmic drug therapy is often constrained by efficacy and safety concerns, and the coexistence of multiple arrhythmia mechanisms frequently complicates treatment [9]. Previous studies have reported suboptimal outcomes of catheter ablation in postoperative CHD patients [10]. However, most available evidence originates from Western countries, and data regarding contemporary ablation practice in Japan remain scarce.

Accordingly, the present study aimed to investigate the current status of catheter ablation for arrhythmias after CHD surgery in Japan and to evaluate its effectiveness and safety in a real‐world, multicenter setting.

## 2 Methods

This multicenter, observational, retrospective study investigated patients who underwent catheter ablation for postoperative arrhythmias associated with congenital heart disease between April 2007 and December 2025. The study protocol was approved by the Institutional Review Board of Saitama Medical University International Medical Center (approval number: 2025–072) through a centralized review process.

### 2.1 Data Collection

This study was a retrospective, registry‐based analysis designed to characterize contemporary catheter ablation practice for arrhythmias following surgery for CHD. We assessed the severity of the underlying CHD, the types and numbers of arrhythmias, the presence of prosthetic valves or surgical patches, and procedural outcomes, including acute success and complication rates.

### 2.2 Definitions and Classification of CHD

CHD severity was classified as mild, moderate, or severe according to the guidelines of the Japanese Circulation Society [11].

### 2.3 Surgical and Anatomical Characteristics

#### 2.3.1 Type of Repair

- Biventricular repair
- Fontan circulation
- Catheter‐based intervention
- Unrepaired CHD
- Palliative surgery only or unclassifiable repair status

#### 2.3.2 Atrial Septal Defect (ASD) Closure

- Direct surgical closure
- Patch closure
- Transcatheter closure

Cases in which the operative records were unavailable, and the method of septal closure could not be confirmed, were classified as unknown.

#### 2.3.3 Fontan Procedures

- Atriopulmonary connection (APC) Fontan
- Lateral tunnel (LT) Fontan
- Intra‐atrial graft Fontan
- Extracardiac conduit Fontan

#### 2.3.4 Atrial Switch Operation for Transposition of the Great Arteries

- Mustard procedure
- Senning procedure

#### 2.3.5 Prosthetic Valve Replacement Characteristics

Valve type: Mechanical or bioprosthetic.

Valve position, classified according to functional anatomy as:

- Functional Pulmonary valve
- Functional Aortic valve
- Functional Systemic atrioventricular valve
- Functional Pulmonary atrioventricular valve

### 2.4 Ablation‐Related Data and Procedural Outcomes

With respect to ablation characteristics, arrhythmia subtype, and the target chamber were identified based on functional anatomy. The type of three‐dimensional electroanatomical mapping system used during the procedure was also recorded.

In cases requiring transseptal or trans‐baffle access, puncture was performed using a long sheath, and detailed data regarding the puncture technique were collected, including the type of puncture needle (wire, radiofrequency needle, or metal needle) and whether the needle traversed prosthetic material or adjacent native tissue [12, 13, 14, 15].

Procedural efficacy was assessed acutely and defined as follows:

- Success:

Complete non‐inducibility of the target arrhythmia.

- Partial success:

Residual inducibility of some arrhythmias.

- Failure:

No change in arrhythmia inducibility.

The type of three‐dimensional mapping system used during the procedure (CARTO, EnSite, Rhythmia, or fluoroscopic mapping) was recorded. Procedure‐related complications were systematically collected and analyzed in detail.

### 2.5 Statistical Analyses

Statistical analyses were performed using JMP Pro, version 18 (SAS Institute), and Python (version 3.11.0). Data distribution was assessed using the Shapiro–Wilk test. Continuous variables are presented as medians with interquartile ranges (IQRs). Non‐normally distributed continuous variables were compared among groups using the Kruskal–Wallis test, followed by Dunn's post hoc test for multiple comparisons. Correlations between variables were evaluated using Spearman's rank correlation coefficient. The association between age and disease severity was assessed using ordinal logistic regression, with age modeled per 10‐year increase. Category‐specific binary logistic regression analyses were additionally performed. Odds ratios (ORs) with 95% confidence intervals (CIs) were reported.

Additional univariable and multivariable logistic regression analyses were performed to explore factors associated with complete procedural success. Complete procedural success was used as the dependent variable, and candidate explanatory variables were selected based on clinical relevance. Adjusted odds ratios (aORs) with 95% confidence intervals were calculated.

All statistical tests were two‐sided, and a *p*‐value < 0.05 was considered statistically significant.

## 3 Results

### 3.1 Patient Characteristics

**FIGURE 1:**
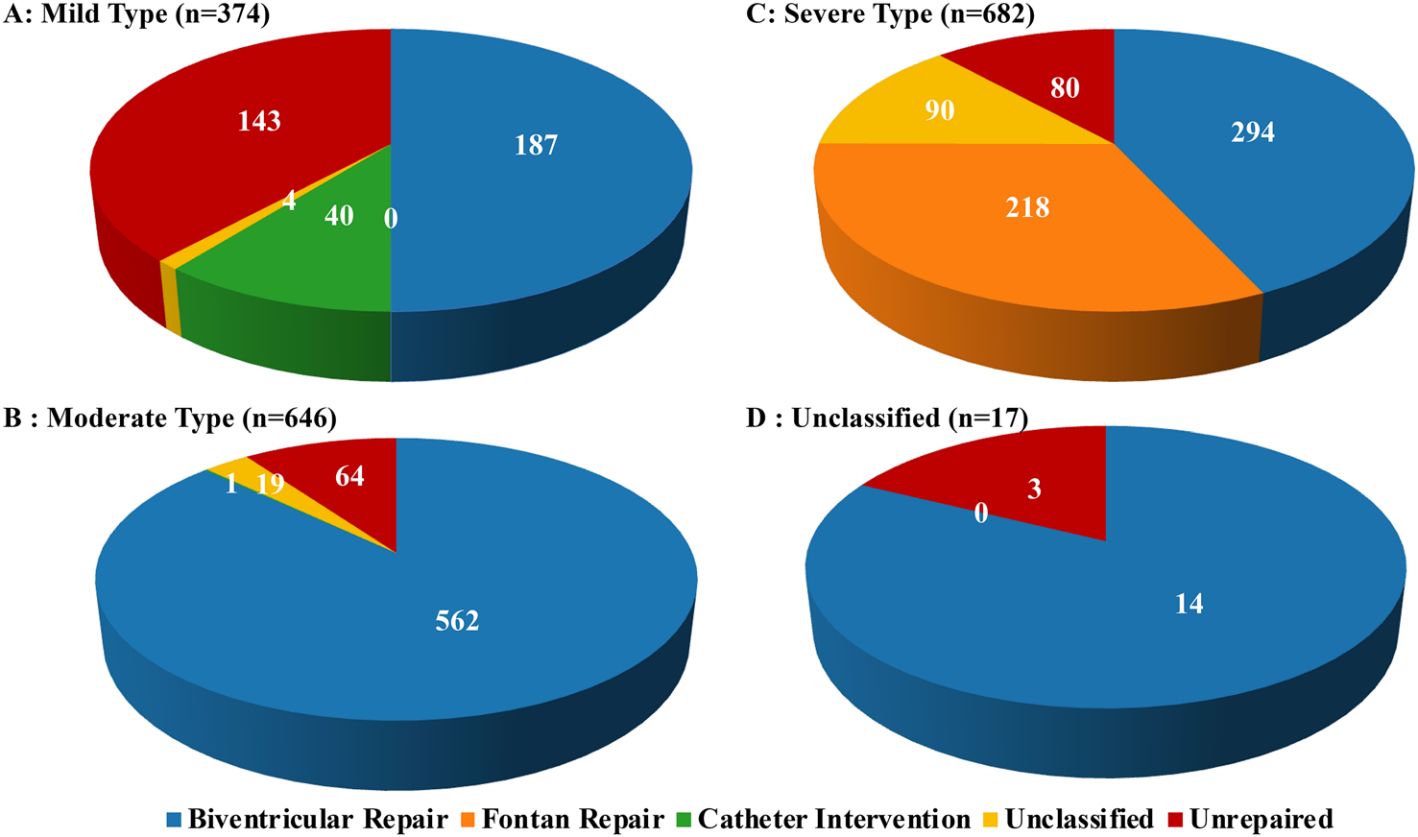
Treatment Strategies and Severity of Congenital Heart Disease. Distribution of treatment strategies according to CHD severity.

A total of 1 719 patients were enrolled from 10 centers. Figure 1 presents the number of patients in each disease severity category, including unclassified cases, along with the corresponding repair methods (Tables S1–S4).

**FIGURE 2:**
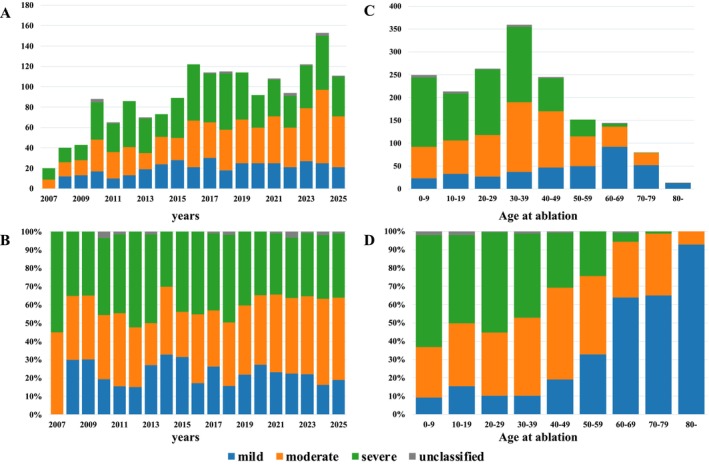
Temporal Trends in Ablation Procedures and Age‐Related Differences. Annual trends in catheter ablation procedures and CHD severity distribution. (A, B) The number of ablation procedures increased over time across all CHD severity categories. (C, D) Increasing age was significantly associated with lower CHD severity.

Figure 2A,B shows the annual number of ablation procedures and the distribution of the severity. The number of ablation cases increased over time regardless of CHD severity (mild, ρ = 0.7020, *p* = 0.0008; moderate, ρ = 0.8920, *p* < 0.0001; severe, ρ = 0.5391, *p* = 0.0172; overall, ρ = 0.8244, *p* < 0.0001) (Supplemental Table 5). As shown in Figure 2C,D, increasing age was significantly associated with lower disease severity (Table S6) In the ordinal logistic regression analysis, each 10‐year increase in age was associated with a significantly lower odds of being classified into a more severe category (OR 0.635 [0.604–0.667], *p* < 0.001). In category‐specific analyses, the proportion of mild cases increased with age (OR 1.649 [1.542–1.763], *p* < 0.001), whereas the proportion of severe cases decreased (OR 0.661 [0.624–0.700], p < 0.001). No significant trend was observed for moderate disease severity (OR 1.039, *p* = 0.116).

**FIGURE 3:**
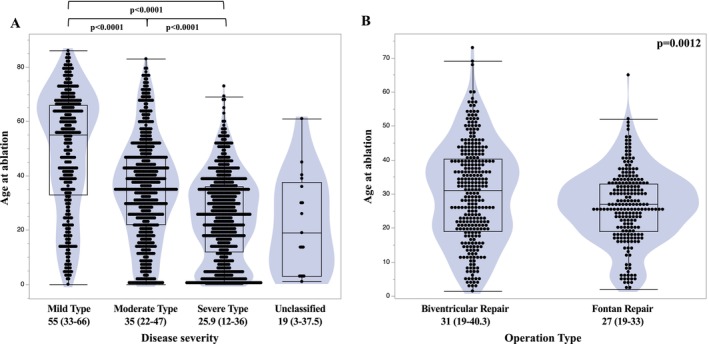
Association Between Disease Severity and Age at Ablation. (A) Relationship between CHD severity and age at the time of ablation, demonstrating a trend toward older age with decreasing disease severity. (B) Among patients with severe CHD, those who underwent a Fontan repair (intracardiac repair) underwent ablation at a significantly younger age compared with those who underwent a biventricular repair.

Figure 3A shows the median age at the time of ablation across CHD severity categories. Patients with more severe CHD tended to undergo ablation at a younger age. Figure 3B presents a comparison of age at ablation in the severe CHD group between patients who underwent a biventricular repair and those who underwent a Fontan repair. Patients who underwent a Fontan repair underwent ablation at a significantly younger age than those who underwent a biventricular repair.

### 3.2 Arrhythmia Details

**FIGURE 4:**
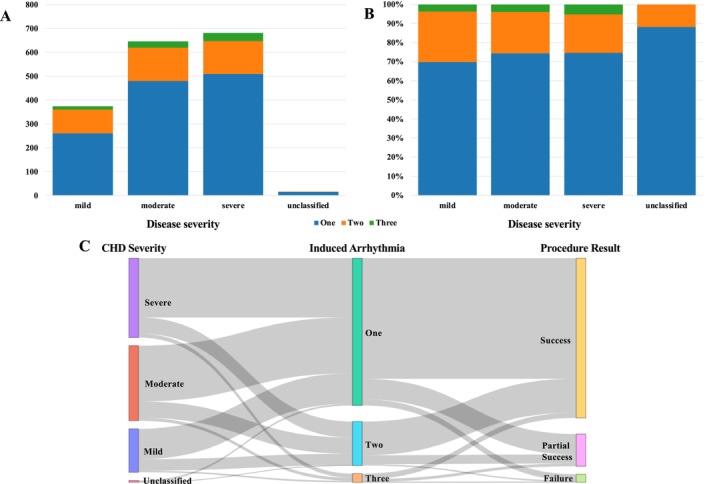
Relationship Between CHD Severity and Induced Arrhythmias. (A, B) Distribution of the number of induced arrhythmia types according to CHD severity. No significant difference was observed among the three severity groups (χ^2^ = 6.63, df = 4, *p* = 0.157). (C) Sankey diagram depicting the flow from CHD severity to the number of induced arrhythmias and procedural outcomes.

Figure 4A,B presents the relationship between CHD severity and the number of induced arrhythmias (Supplemental Table 7). The distribution of the number of arrhythmia types did not differ significantly among the three disease severity groups (χ^2^ = 6.63, df = 4, *p* = 0.157). Figure 4C shows the Sankey diagram of the disease severity, the number of induced arrhythmias, and the procedure results. The relationship between the number of arrhythmia types and acute procedural success was examined. The distribution of acute procedural outcomes (success, partial success, and failure) differed significantly according to the number of arrhythmia types (χ^2^ = 24.77, df = 4, *p* < 0.0001). Success rates decreased as the number of arrhythmia types increased, from 81.9% in patients with one arrhythmia type to 76.5% in those with two types and 62.7% in those with three types. A Cochran–Armitage trend test demonstrated a significant monotonic decrease in the rate of complete success with increasing numbers of arrhythmia types (χ^2^ = 17.88, *p* < 0.0001). In logistic regression analysis, each additional arrhythmia type was associated with a significantly lower likelihood of complete success (OR 0.66 [0.54–0.80]; *p* < 0.0001).

**FIGURE 5:**
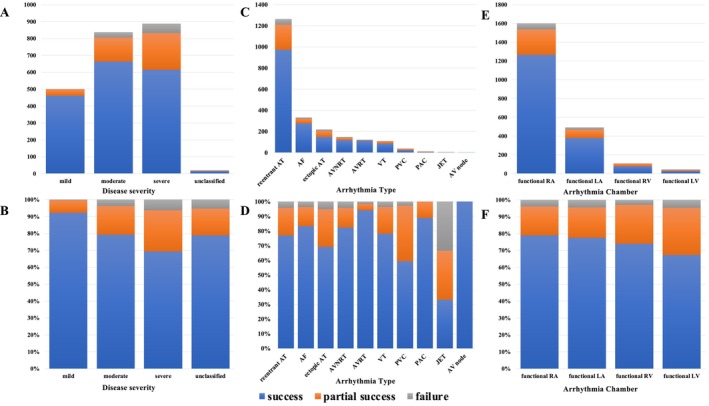
Procedural Outcomes According to CHD Severity, Arrhythmia Type, and Target Chamber. (A, B) Acute procedural outcomes stratified by CHD severity. Complete procedural success decreased stepwise with increasing disease severity. (C, D) Distribution of induced arrhythmia types and corresponding procedural outcomes. Reentrant atrial tachycardia was the most common arrhythmia, with the highest complete success observed in atrioventricular reentrant tachycardia and the lowest in premature ventricular contractions. (E, F) Procedural outcomes according to the targeted arrhythmia chamber, with the functional right atrium being the most frequently treated chamber.

Figure 5A,B summarizes the procedural outcomes according to CHD severity (Supplemental Table 8). Acute procedural outcomes differed significantly across severity categories (mild, moderate, and severe) (χ^2^ = 71.54, df = 4, *p* < 0.0001). Complete success rates decreased stepwise with increasing disease severity, from 92.5% in the mild group to 81.4% in the moderate group and 71.4% in the severe group. A significant association with disease severity was observed when acute success was classified as complete or non‐complete success (χ^2^ = 68.47, df = 2, *p* < 0.0001). A Cochran–Armitage trend test further demonstrated a significant monotonic decrease in complete success with increasing disease severity (χ^2^ = 68.40, df = 1, *p* < 0.0001). In logistic regression analysis using mild disease as the reference category, moderate disease (OR 0.35[CI, 0.23–0.55]; *p* < 0.0001) and severe disease (OR 0.20 [0.13–0.31]; p < 0.0001) were independently associated with a lower likelihood of complete procedural success.

Figure 5C,D shows the types of induced arrhythmias and the associated procedural outcomes (Supplemental Tables 9 and 10). Reentrant AT was the most frequently observed arrhythmia. Excluding three cases of junctional ectopic tachycardia and one case of atrioventricular node ablation, the complete procedural success rate was highest for atrioventricular reentrant tachycardia and lowest for premature ventricular contractions. Figure 5E,F presents the relationship between the arrhythmia chamber and procedural outcomes, with the functional RA being the most common chamber targeted for treatment.

**FIGURE 6:**
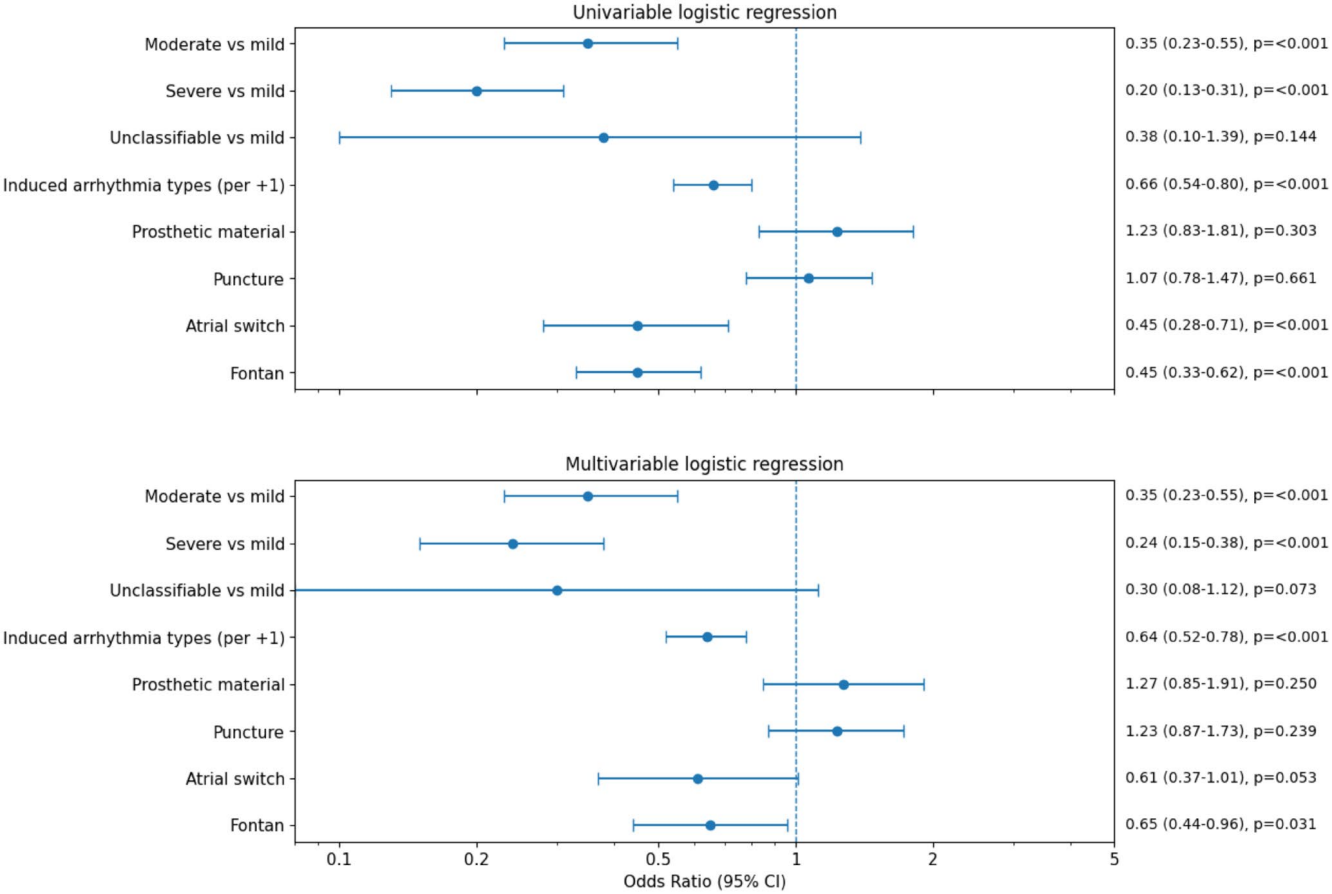
Univariable and multivariable logistic regression analyses of CHD‐related factors associated with complete procedural success. Forest plots showing odds ratios and 95% confidence intervals for clinical and procedural factors associated with complete procedural success. Variables included Fontan surgery, number of induced arrhythmia types, prosthetic material, puncture status, CHD severity, and atrial switch repair. Mild CHD was used as the reference category for severity.

Figure 6 shows the forest plot of the success. In univariable logistic regression analysis, Fontan surgery, a greater number of induced arrhythmia types, higher CHD severity, and atrial switch repair were associated with lower odds of complete procedural success, whereas prosthetic material and puncture status were not significantly associated with procedural success. In multivariable logistic regression analysis with complete procedural success as the dependent variable, moderate CHD severity (adjusted odds ratio [aOR] 0.35, 95% confidence interval [CI] 0.23–0.55, *p* < 0.001), severe CHD severity (aOR 0.24, 95% CI 0.15–0.38, *p* < 0.001), Fontan surgery (aOR 0.65, 95% CI 0.44–0.96, *p* = 0.031), and a greater number of induced arrhythmia types (aOR 0.64 per additional arrhythmia type, 95% CI 0.52–0.78, *p* < 0.001) were independently associated with lower odds of complete procedural success.

Procedure‐related complications occurred in 39 patients (2.3%) (mild CHD, *n* = 7 [0.4%]; moderate CHD, *n* = 11 [0.6%]; severe CHD, *n* = 21 [1.2%]; unclassified CHD, *n* = 0 [0%]) (Supplemental Table 11). Excluding seven cases with access‐site complications, the most frequent complication was worsening heart failure, defined as the development of symptomatic left‐ and/or right‐sided heart failure (*n* = 6 [0.35%]). Pericardial effusion was observed in three patients (0.17%), of whom one required pericardial drainage and one required surgical intervention. Cerebral infarction was rare, with only one case of transient ischemic attack in a patient who underwent ablation for reentrant AT of the RA and AF.

**FIGURE 7:**
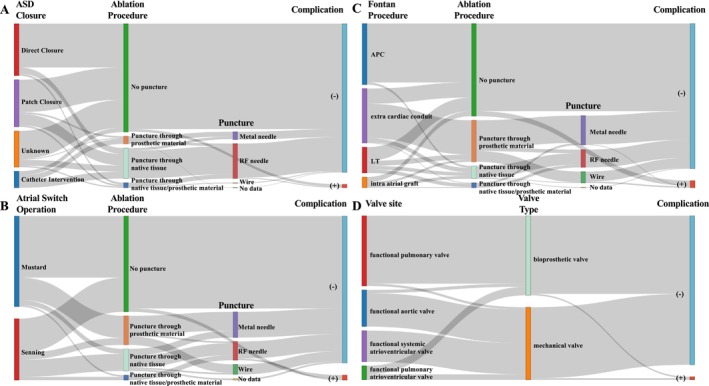
Surgical Repair Type, Access Approach, and Procedural Complications. Sankey diagrams illustrating the relationships among surgical repair type, access approach, and procedural complications in patients after atrial septal defect repair (A), atrial switch operation (B), Fontan surgery (C), and prosthetic valve replacement (D). Procedural complications occurred in 8 patients (2.3%) after an atrial septal defect repair, 3 patients (3.4%) after an atrial switch operation, 10 patients (4.7%) after Fontan surgery, and 4 patients (2.1%) after prosthetic valve replacement.

Figure 7 presents Sankey diagrams illustrating the relationships among surgical repair type, access approach, and procedural complications in patients after ASD repair (A), atrial switch operation (B), Fontan surgery (C), and prosthetic valve replacement (D). A total of 348 patients underwent ASD closure. Among them, 8 patients (2.3%) experienced complications (Supplemental Table 12). A Fontan operation was performed in 216 patients, including 4 patients who underwent a Björk procedure. Complications occurred in 10 patients (4.6%) in this group (Table S13). Atrial switch operations were performed in 89 patients, with complications observed in 3 patients (3.4%) (Table S14). Prosthetic valve replacements were performed in 195 patients (Table S15), and 4 patients (2.1%) developed complications (Table S16).

Figure 7 also illustrates the relationship between the puncture method and the occurrence of complications. Among patients with a prior ASD closure, a puncture was performed in 100 patients, and complications occurred in 2 (2.0%) (Table S17). Among patients with a prior atrial switch operation, 33 underwent a puncture, and 1 patient (3.0%) developed a complication (Supplemental Table 18). In patients who had previously undergone the Fontan procedure, a puncture was performed in 83 patients, with complications observed in 3 patients (3.6%) (Supplemental Table 19). Among the 216 patients who underwent a puncture, pericardial effusion–related complications were observed in 2 patients (0.9%).

The mapping systems used for ablation were CARTO in 1 312 cases, EnSite in 266 cases, Rhythmia in 119 cases, and fluoroscopic guidance in 22 cases. Fluoroscopic guidance was predominantly used in younger patients, whereas with increasing age, EnSite, CARTO, and Rhythmia were used in that order (Figure S1).

## 4 Discussion

### 4.1 Major Findings

The major findings of this study can be summarized as follows:

1. The number of ablation procedures performed in patients after CHD surgery has increased over time.
2. Ablation procedures were more frequently performed in patients with severe CHD at younger ages. Among patients with severe CHD, ablation was performed at a younger age after a Fontan repair than after a biventricular repair.
3. Procedural success rates decreased as the number of induced arrhythmia types increased, and success rates were lower in arrhythmias associated with severe CHD.
4. The overall complication rate was 2.3%, and excluding vascular complications, worsening heart failure was the most common adverse event. Among patients who required puncture after ASD closure, atrial switch operation, or Fontan surgery, pericardial effusion–related complications occurred in 2 patients (0.9%).

### 4.2 Ablation Trends in the Number of Ablation Procedures in Patients With CHD

Advances in the treatment of CHD have led to improved clinical outcomes and long‐term survival in recent years. Consequently, the need for catheter ablation to treat arrhythmias associated with CHD has increased. According to data from the J‐AB registry, although the proportion of ablation procedures performed in patients with CHD has remained relatively stable among all ablation cases, the absolute number of such procedures has shown an increasing trend [16, 17, 18, 19]. However, detailed information regarding patient characteristics and procedural aspects of CHD‐related ablation has remained limited. The present study demonstrates that the number of ablation procedures in patients with CHD has increased over time. Furthermore, the present study demonstrates that reentrant AT was the most common arrhythmia subtype among CHD‐related ablation procedures.

Previous reports focusing on AF in patients with CHD have predominantly included individuals with mild CHD [20]. In contrast, our study analyzed all CHD‐related ablation procedures and included a substantial proportion of patients with severe CHD. The higher prevalence of severe CHD in our cohort may explain the predominance of reentrant AT, as multiple prior surgeries and surgical scars likely serve as arrhythmogenic substrates facilitating reentrant circuits.

In the present study, a higher proportion of ablation procedures in elderly patients was associated with mild CHD (Figure 1D); however, the absolute number of ablation procedures performed in elderly patients remained low (Figure 1C). With further improvements in long‐term survival among patients with moderate to severe CHD, driven by advances in medical therapy, age‐related arrhythmogenic substrate changes may increasingly contribute to arrhythmia development [9, 21]. As a result, both the number of ablation procedures and the underlying disease characteristics may change over time. These findings suggest a potential need for a broader range of electrophysiologists to acquire expertise in catheter ablation for arrhythmias associated with CHD.

### 4.3 Ablation Outcomes According to CHD Severity

Recent advances in ablation technology have been reported to improve procedural outcomes, with acute procedural success rates increasing to 87%–97%, depending on CHD type, over the past 20 years [22, 23, 24, 25]. The evolution of three‐dimensional mapping systems has enabled high‐density mapping, allowing more precise delineation of arrhythmia mechanisms [26, 27, 28]. This has facilitated the identification of arrhythmia circuits involving residual potentials within scar tissue, thereby contributing to improved treatment outcomes. In addition, advances in ablation technologies, including the introduction of contact force–sensing catheters and lesion quality indices, have enabled the creation of more durable lesions and may have further improved procedural success [29].

Nevertheless, our study demonstrated that ablation success rates decreased with increasing CHD severity (Figure 5B). In patients with severe CHD, multiple prior surgeries and complex surgical incisions and sutures may give rise to multiple arrhythmogenic circuits. Furthermore, CHD‐specific substrates, such as twin atrioventricular nodes, may also contribute to the observed reduction in procedural success.

### 4.4 Procedural Complications of CHD Ablation

In the present study, the overall complication rate of catheter ablation in patients with CHD was 2.3%. According to the J‐AB registry, the overall complication rates for all ablation procedures in recent years were reported to be 2.27% in 2022 and 2.35% in 2021, indicating that the complication rate of CHD‐associated ablation is comparable to that of ablation procedures in the general population [16, 17]. Pericardial effusion occurred in three patients, of whom two required treatment for cardiac tamponade (0.12%). The incidence of cardiac tamponade was lower than that reported for all ablation procedures in the J‐AB registry. Furthermore, even when the analysis was limited to patients who required a puncture procedure and had undergone prior Fontan surgery, ASD closure, or atrial switch operation, cardiac tamponade occurred in only 2 cases (0.9%). The reported incidence of cardiac tamponade during AF ablation ranges from 0.5% to 1.2% [16, 19, 30]. In this context, the safety profile observed in our cohort appears to be within an acceptable range.

In contrast, worsening heart failure was the second most frequent complication, which is a distinctive finding of this study. In patients with CHD, baseline hemodynamics often differ from those in patients without structural heart disease. Prolonged ablation procedures and changes in shunt flow related to vascular access may result in postoperative hemodynamic deterioration, potentially increasing the risk of heart failure. Therefore, catheter ablation in patients with CHD may require particularly careful postprocedural management.

## 5 Limitations

This study has several limitations. First, the present analysis focused on acute procedural outcomes, and long‐term success rates were not evaluated. Consequently, the procedural performance and clinical outcomes in patients with arrhythmia recurrence remained beyond the scope of this study. Previous studies have reported relatively lower ablation success rates in patients with CHD, and long‐term outcomes may be further reduced [10]. Second, procedural outcomes were compared according to arrhythmia type; therefore, more detailed analyses based on underlying cardiac anatomy and arrhythmia mechanisms are warranted. Third, because this study included ablation procedures performed since 2007, advances in ablation technology over time may have influenced procedural success rates. Finally, this study collected ablation data from well‐experienced centers for CHD ablation in Japan. As a result, the proportion of moderate to severe CHD was higher than that of mild CHD in this registry. Therefore, our findings may predominantly reflect practice patterns at such specialized centers. Larger multinational registries will be needed to better characterize global practice patterns and outcomes of CHD‐related ablation.

## 6 Conclusion

Catheter ablation for postoperative CHD‐related arrhythmias is increasingly performed in Japan and can be achieved with acceptable safety. However, procedural success decreases with greater disease severity and arrhythmia burden, emphasizing the need for disease‐specific strategies and specialized expertise.

## Author Contributions

H.M., A.M., T.S., S.T., J.Y., M.S., and N.S. study conception, data collection, and design; N.N., Y.K., T.K., H.A., S.H., K.N., K.T., D.T., T.S., K.T., R.H., M.M., D.Y., S.H., Y.M., S.W., K.K., K.K., Y.H., T.M., K.M., M.N., W.S., T.N., K.N., and K.T. contributed to the data collection, manuscript revision, and data analysis; T.K., R.K., M.S., and N.S. provided the study supervision.

## Disclosure

H.M. received lecture fees from Biosense Webster Japan and Boston Scientific Japan. Our department received grant support from Boston Scientific Japan and Abbott Medical Japan.

## Ethics Statement

The study protocol was approved by the hospital's institutional review board (IRB No. 2025–072).

## Conflicts of Interest

The authors declare no conflicts of interest.

## Supporting information


**Figure S1:** Mapping Systems Used for Catheter Ablation According to Age.
Distribution of mapping systems used for catheter ablation procedures. CARTO was used in 1 312 cases, EnSite in 266 cases, Rhythmia in 119 cases, and fluoroscopic guidance in 22 cases. Fluoroscopic guidance was predominantly used in younger patients, whereas with increasing age, EnSite, CARTO, and Rhythmia were used in that order.

**Table S1:** Background CHD characteristics (Mild Type).
**Table S2:** Background CHD characteristics (Moderate Type).
**Table S3:** Background CHD characteristics (Severe Type).
**Table S4:** Background CHD characteristics (Unclassified).
**Table S5:** Procedural year and CHD severity.
**Table S6:** Procedural age and CHD severity.
**Table S7:** Number of induced arrhythmias and CHD severity.
**Table S8:** CHD severity and procedure results (per arrhythmia).
**Table S9:** Arrhythmia type and procedure results.
**Table S10:** Arrhythmia chamber and procedure results.
**Table S11:** Details of complications in all patients.
**Table S12:** Details of complications in patients with an ASD closure operation.
**Table S13:** Details of complications in patients with a Fontan operation.
**Table S14:** Details of complication in patients with an Atrial Switch operation.
**Table S15:** Incidence of Ablation Complications After Prosthetic Valve Replacement.
**Table S16:** Details of complications in patients with valve surgery.
**Table S17a:** Puncture Methods and complication rates in postoperative ASD patients (direct closure).
**Table S17b:** Puncture methods and complication rates in postoperative ASD patients (patch closure).
**Table S17c:** Puncture methods and complication rates in postoperative ASD patients (catheter intervention).
**Table S17d:** Puncture methods and complication rates in postoperative ASD patients (unknown).
**Table S18a:** Puncture methods and complication rates in postoperative atrial switch patients (mustard).
**Table S18b:** Puncture methods and complication rates in postoperative atrial switch patients (Senning).
**Table S19a:** Puncture methods and complication rates in postoperative Fontan patients (APC Fontan).
**Table S19b:** Puncture methods and complication rates in postoperative Fontan patients (extracardiac conduit).
**Table S19c:** Puncture methods and complication rates in postoperative Fontan patients (intra‐atrial graft).
**Table S19d:** Puncture methods and complication rates in postoperative Fontan patients (lateral tunnel).

## Data Availability

The data that support the findings of this study are available on request from the corresponding author. The data are not publicly available due to privacy or ethical restrictions.
