# Supplementary figures and images for: REgistry Of Catheter AbLation After Congenital Heart Disease Surgery(REAL‐CHD Registry)
Source: J Arrhythm. 2026 Apr 20;42(2):e70347. doi: 10.1002/joa3.70347 (PMC13093813; doi:10.1002/joa3.70347)

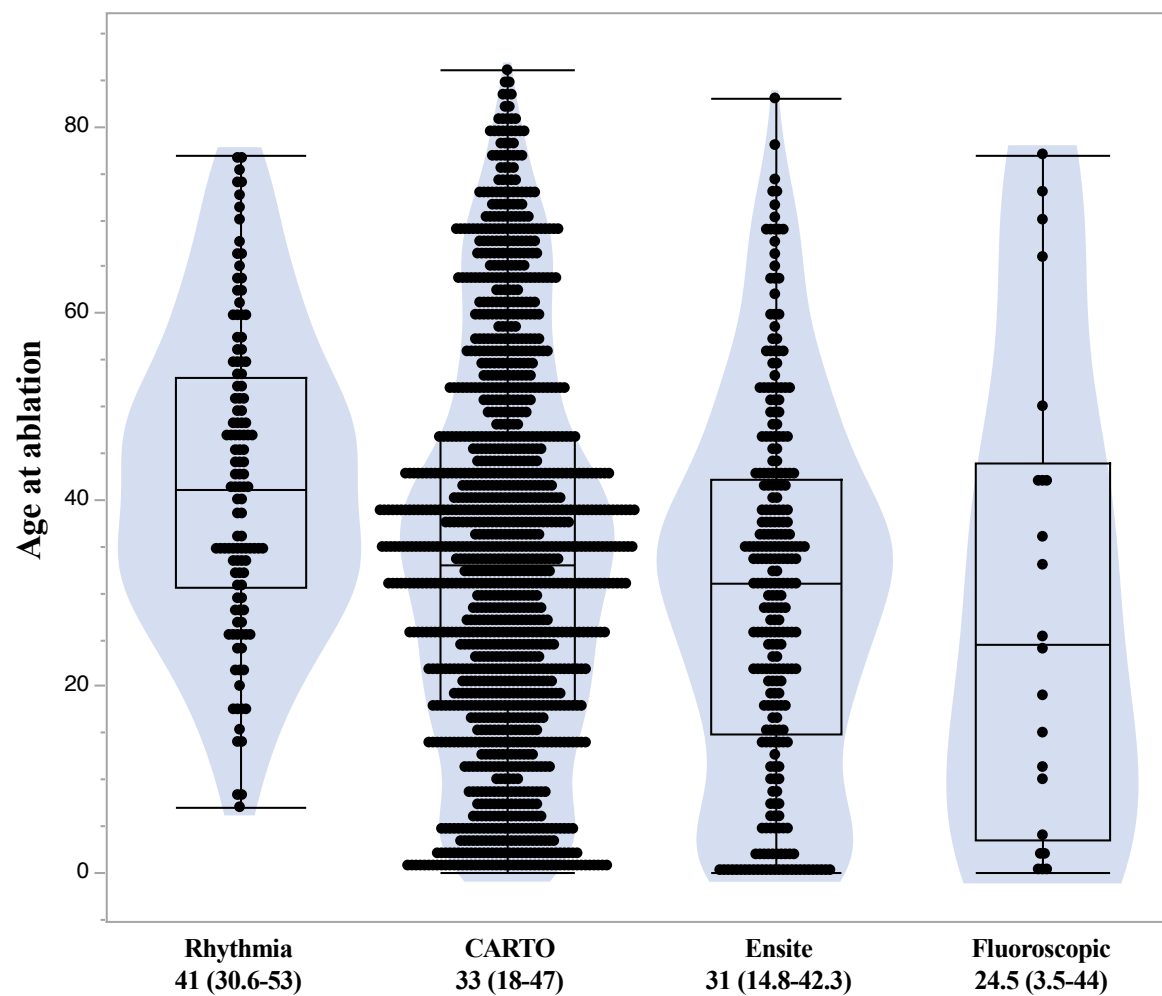

Supplement: Supplementary file 1 — Figure S1: Mapping Systems Used for Catheter Ablation According to Age. Distribution of mapping systems used for catheter ablation procedures. CARTO was used in 1 312 cases, EnSite in 266 cases, Rhythmia in 119 cases, and fluoroscopic guidance in 22 cases. Fluoroscopic guidance was predominantly used in younger patients, whereas with increasing age, EnSite, CARTO, and Rhythmia were used in that order. [file JOA3-42-e70347-s001.pdf]
