# Supplementary material for: REgistry Of Catheter AbLation After Congenital Heart Disease Surgery(REAL‐CHD Registry)
Source: J Arrhythm. 2026 Apr 20;42(2):e70347. doi: 10.1002/joa3.70347 (PMC13093813; doi:10.1002/joa3.70347)
Supplement: Supplementary file 2 — Table S1: Background CHD characteristics (Mild Type). Table S2: Background CHD characteristics (Moderate Type). Table S3: Background CHD characteristics (Severe Type). Table S4: Background CHD characteristics (Unclassified). Table S5: Procedural year and CHD severity. Table S6: Procedural age and CHD severity. Table S7: Number of induced arrhythmias and CHD severity. Table S8: CHD severity and procedure results (per arrhythmia). Table S9: Arrhythmia type and procedure results. Table S10: Arrhythmia chamber and procedure results. Table S11: Details of complications in all patients. Table S12: Details of complications in patients with an ASD closure operation. Table S13: Details of complications in patients with a Fontan operation. Table S14: Details of complication in patients with an Atrial Switch operation. Table S15: Incidence of Ablation Complications After Prosthetic Valve Replacement. Table S16: Details of complications in patients with valve surgery. Table S17a: Puncture Methods and complication rates in postoperative ASD patients (direct closure). Table S17b: Puncture methods and complication rates in postoperative ASD patients (patch closure). Table S17c: Puncture methods and complication rates in postoperative ASD patients (catheter intervention). Table S17d: Puncture methods and complication rates in postoperative ASD patients (unknown). Table S18a: Puncture methods and complication rates in postoperative atrial switch patients (mustard). Table S18b: Puncture methods and complication rates in postoperative atrial switch patients (Senning). Table S19a: Puncture methods and complication rates in postoperative Fontan patients (APC Fontan). Table S19b: Puncture methods and complication rates in postoperative Fontan patients (extracardiac conduit). Table S19c: Puncture methods and complication rates in postoperative Fontan patients (intra‐atrial graft). Table S19d: Puncture methods and complication rates in postoperative Fontan patients (lateral tunnel). [file JOA3-42-e70347-s002.docx]

| Mild Type | **Number (n=374)** |
| --- | --- |
| Atrial septal defect (secundum or sinus venosus type, no residual lesion, repaired) | 133 (35.6%) |
| Atrial septal defect (small, isolated, unrepaired) | 130 (34.8%) |
| Ventricular septal defect (no residual lesion, repaired) | 78 (20.9%) |
| Ventricular septal defect (small, isolated, unrepaired) | 14 (3.7%) |
| Patent ductus arteriosus (repaired) | 8 (2.1%) |
| Mitral valve disease (isolated, unrepaired) | 4 (1.1%) |
| Aortic valve disease (isolated, bicuspid valve, unrepaired) | 4 (1.1%) |
| Tricuspid regurgitation | 1 (0.3%) |
| Patent ductus arteriosus (mild, unrepaired) | 1 (0.3%) |
| Pulmonary stenosis (mild, unrepaired) | 1 (0.3%) |

**Supplemental Table 1. Background CHD characteristics (Mild Type)**

| Moderate Type | **Number (n=646)** |
| --- | --- |
| Tetralogy of Fallot (repaired) | 315 (48.8%) |
| Ebstein anomaly | 97 (15.0%) |
| Complete or partial atrioventricular septal defect | 54 (8.4%) |
| Complete transposition of the great arteries (after arterial switch) | 47 (7.3%) |
| Total or partial anomalous pulmonary venous return | 35 (5.4%) |
| Coarctation of the aorta | 18 (2.8%) |
| Pulmonary valve stenosis (moderate or greater) | 16 (2.5%) |
| Aortic stenosis (sub valvular or supravalvular) | 15 (2.3%) |
| Ventricular septal defect with right ventricular outflow tract obstruction | 9 (1.4%) |
| Ventricular septal defect with mitral valve disease | 7 (1.1%) |
| Right ventricular outflow tract stenosis | 6 (0.9%) |
| Ventricular septal defect with subaortic stenosis | 6 (0.9%) |
| Anomalous origin of the coronary artery (including ALCAPA) | 5 (0.8%) |
| Ventricular septal defect with aortic regurgitation | 5 (0.8%) |
| Atrial septal defect (primum type or large unrepaired defect) | 4 (0.6%) |
| Ventricular septal defect with coarctation of the aorta | 3 (0.5%) |
| Atrial septal defect (sinus venosus type) | 3 (0.5%) |
| Marfan syndrome or Turner syndrome | 1 (0.2%) |

**Supplemental Table 2. Background CHD characteristics (Moderate Type)**

| Severe Type | **Number (n=682)** |
| --- | --- |
| Double outlet right ventricle or double outlet left ventricle | 112 (16.4%) |
| Congenitally corrected transposition of the great arteries | 109 (16.0%) |
| Single ventricle physiology | 82 (12.0%) |
| Status post Fontan procedure | 76 (11.1%) |
| Complete transposition of the great arteries (excluding arterial switch) | 72 (10.6%) |
| Pulmonary atresia | 67 (9.8%) |
| All unrepaired or palliated cyanotic congenital heart diseases | 58 (8.5%) |
| Atrioventricular and ventriculoarterial discordance | 49 (7.2%) |
| Tricuspid atresia | 24 (3.5%) |
| Status post conduit repair | 15 (2.2%) |
| Residual truncus arteriosus | 7 (1.0%) |
| Mitral atresia | 4 (0.6%) |
| Pulmonary arterial hypertension / Eisenmenger syndrome | 4 (0.6%) |
| Interrupted aortic arch | 3 (0.4%) |

**Supplemental Table 3. Background CHD characteristics (Severe Type)**

| Unclassified | **Number (n=17)** |
| --- | --- |
| Congenital mitral regurgitation; status post MVR/MVP | 7 (41.2%) |
| Cor triatriatum | 2 (11.8%) |
| Status post cardiac tumor resection | 1 (5.9%) |
| Congenital aortic stenosis | 1 (5.9%) |
| Congenital tricuspid regurgitation | 1 (5.9%) |
| Bicuspid aortic valve; status post AVR | 1 (5.9%) |
| Mitral valve prolapse | 1 (5.9%) |
| Congenital aortic stenosis/mitral regurgitation; status post AVP/MVR | 1 (5.9%) |
| Congenital mitral regurgitation | 1 (5.9%) |
| Uhl’s anomaly | 1 (5.9%) |

**Supplemental Table 4. Background CHD characteristics (Unclassified)**

| **Year** | **Mild** | **Moderate** | **Severe** | **Unclassified** |
| --- | --- | --- | --- | --- |
| **2007 (n=20)** | **0 (0%)** | **9 (45.0%)** | **11 (55.0%)** | **0 (0%)** |
| **2008 (n=40)** | **12 (30.0%)** | **14 (35.0%)** | **14 (35.0%)** | **0 (0%)** |
| **2009 (n=43)** | **13 (30.2%)** | **15 (34.9%)** | **15 (34.9%)** | **0 (0%)** |
| **2010 (n=88)** | **17 (19.3%)** | **31 (35.2%)** | **37 (42.0%)** | **3 (3.4%)** |
| **2011 (n=65)** | **10 (15.4%)** | **26 (40.0%)** | **28 (43.1%)** | **1 (1.5%)** |
| **2012 (n=86)** | **13 (15.1%)** | **28 (32.6%)** | **45 (52.3%)** | **0 (0%)** |
| **2013 (n=70)** | **19 (27.1%)** | **16 (22.9%)** | **34 (48.6%)** | **1 (1.4%)** |
| **2014 (n=73)** | **24 (32.9%)** | **27 (37.0%)** | **22 (30.1%)** | **0 (0%)** |
| **2015 (n=89)** | **28 (31.5%)** | **22 (24.7%)** | **39 (43.8%)** | **0 (0%)** |
| **2016 (n=122)** | **21 (17.2%)** | **46 (37.7%)** | **55 (45.1%)** | **0 (0%)** |
| **2017 (n=114)** | **30 (26.3%)** | **35 (30.7%)** | **48 (42.1%)** | **1 (0.9%)** |
| **2018 (n=115)** | **18 (15.7%)** | **40 (34.8%)** | **55 (47.8%)** | **2 (1.7%)** |
| **2019 (n=114)** | **25 (21.9%)** | **43 (37.7%)** | **46 (40.4%)** | **0 (0%)** |
| **2020 (n=92)** | **25 (27.2%)** | **35 (38.0%)** | **32 (34.8%)** | **0 (0%)** |
| **2021 (n=108)** | **25 (23.1%)** | **46 (42.6%)** | **36 (33.3%)** | **1 (0.9%)** |
| **2022 (n=94)** | **21 (22.3%)** | **39 (41.5%)** | **31 (33.0%)** | **3 (3.2%)** |
| **2023 (n=122)** | **27 (22.1%)** | **52 (42.6%)** | **42 (34.4%)** | **1 (0.8%)** |
| **2024 (n=153)** | **25 (16.3%)** | **72 (47.1%)** | **53 (34.6%)** | **3 (2.0%)** |
| **2025 (n=111)** | **21 (18.9%)** | **50 (45.0%)** | **39 (35.1%)** | **1 (0.9%)** |
| **Total (n=1719)** | **374 (21.8%)** | **646 (37.6%)** | **682 (39.7%)** | **17 (1.0%)** |

**Supplemental Table 5. Procedural year and CHD severity**

| **Year** | **mild** | **moderate** | **severe** | **unclassified** |
| --- | --- | --- | --- | --- |
| **0-9 (n=249)** | **23 (9.2%)** | **69 (27.7%)** | **152 (61.0%)** | **5 (2.0%)** |
| **10-19 (n=215)** | **33 (15.3%)** | **75 (34.9%)** | **103 (47.9%)** | **4 (1.9%)** |
| **20-29 (n=263)** | **27 (10.3%)** | **91 (34.6%)** | **144 (54.8%)** | **1 (0.4%)** |
| **30-39 (n=358)** | **37 (10.3%)** | **151 (42.2%)** | **166 (46.4%)** | **4 (1.1%)** |
| **40-49 (n=244)** | **47 (19.3%)** | **123 (50.4%)** | **72 (29.5%)** | **2 (0.8%)** |
| **50-59 (n=152)** | **50 (32.9%)** | **65 (42.8%)** | **37 (24.3%)** | **0 (0%)** |
| **60-69 (n=144)** | **92 (63.9%)** | **44 (30.6%)** | **7 (4.9%)** | **1 (0.7%)** |
| **70-79 (n=80)** | **52 (65.0%)** | **27 (33.8%)** | **1 (1.3%)** | **0 (0%)** |
| **80- (n=14)** | **13 (92.9%)** | **1 (7.1%)** | **0 (0%)** | **0 (0%)** |
| **Total (n=1719)** | **374 (21.8%)** | **646 (37.6%)** | **682 (39.7%)** | **17 (1.0%)** |

**Supplemental Table 6. Procedural age and CHD severity**

| **Number of Arrhythmia** | **mild** | **moderate** | **severe** | **unclassified** |
| --- | --- | --- | --- | --- |
| **One (n=1265)** | **261 (20.6%)** | **480 (37.9%)** | **509 (40.2%)** | **15 (1.2%)** |
| **Two (n=379)** | **99 (26.1%)** | **140 (36.9%)** | **138 (36.4%)** | **2 (0.5%)** |
| **Three (n=75)** | **14 (18.7%)** | **26 (34.7%)** | **35 (46.7%)** | **0 (0%)** |
| **Total (n=1719)** | **374 (21.8%)** | **646 (37.6%)** | **682 (39.7%)** | **17 (1.0%)** |

**Supplemental Table 7. Number of induced arrhythmias and CHD severity**

| **CHD severity** | **success** | **partial success** | **failure** |
| --- | --- | --- | --- |
| **Mild (n=500)** | **461 (92.2%)** | **38 (7.6%)** | **1 (0.2%)** |
| **Moderate (n=837)** | **664 (79.3%)** | **142 (17.0%)** | **31 (3.7%)** |
| **Severe (n=888)** | **616 (69.4%)** | **216 (24.3%)** | **56 (6.3%)** |
| **Unclassified (n=19)** | **15 (78.9%)** | **3 (15.8%)** | **1 (5.3%)** |
| **Total (n=2244)** | **1756 (78.3%)** | **399 (17.8%)** | **89 (4.0%)** |

**Supplemental Table 8. CHD severity and procedure results (per arrhythmia)**

| **Arrhythmia Type** | **success** | **partial success** | **failure** |
| --- | --- | --- | --- |
| **reentrant AT (n=1264)** | **974 (77.0%)** | **238 (18.8%)** | **52 (4.1%)** |
| **AF (n=334)** | **278 (83.2%)** | **44 (13.2%)** | **12 (3.6%)** |
| **ectopic AT (n=218)** | **151 (69.3%)** | **56 (25.7%)** | **11 (5.0%)** |
| **AVNRT (n=147)** | **121 (82.3%)** | **20 (13.6%)** | **6 (4.1%)** |
| **AVRT (n=121)** | **114 (94.2%)** | **5 (4.1%)** | **2 (1.7%)** |
| **VT (n=110)** | **86 (78.2%)** | **20 (18.2%)** | **4 (3.6%)** |
| **PVC (n=37)** | **22 (59.5%)** | **14 (37.8%)** | **1 (2.7%)** |
| **PAC (n=9)** | **8 (88.9%)** | **1 (11.1%)** | **0 (0%)** |
| **JET (n=3)** | **1 (33.3%)** | **1 (33.3%)** | **1 (33.3%)** |
| **AV node (n=1)** | **1 (100%)** | **0 (0%)** | **0 (0%)** |
| **Total (n=2244)** | **1756 (78.3%)** | **399 (17.8%)** | **89 (4.0%)** |

**Supplemental Table 9. Arrhythmia type and procedure results**

| **Arrhythmia chamber** | **success** | **partial success** | **failure** |
| --- | --- | --- | --- |
| **functional RA (n=1601)** | **1265 (79.0%)** | **274 (17.1%)** | **62 (3.9%)** |
| **functional LA (n=492)** | **382 (77.6%)** | **88 (17.9%)** | **22 (4.5%)** |
| **functional RV (n=108)** | **80 (74.1%)** | **25 (23.1%)** | **3 (2.8%)** |
| **functional LV (n=43)** | **29 (67.4%)** | **12 (27.9%)** | **2 (4.7%)** |

**Supplemental Table 10. Arrhythmia chamber and procedure results**

| **Complication Details (n=39 [2.3%])** | | | |
| --- | --- | --- | --- |
| Complication type | Number | Complication type | Number |
| Vascular complication | 7 (0.41%) | urinary tract bleeding | 1 (0.06%) |
| Heart failure worsening | 6 (0.35%) | pericardial effusion | 1 (0.06%) |
| transient AVB | 4 (0.23%) | lead dislodgement | 1 (0.06%) |
| sick sinus syndrome | 3 (0.17%) | hypotension | 1 (0.06%) |
| phrenic nerve paralysis | 3 (0.17%) | ASD laceration | 1 (0.06%) |
| nasal bleeding | 2 (0.12%) | Gastric distension | 1 (0.06%) |
| complete AVB | 2 (0.12%) | cardiac tamponade needed operation | 1 (0.06%) |
| TIA & phrenic nerve paralysis | 1 (0.06%) | cardiac tamponade needed drainage | 1 (0.06%) |
| Gastric distension & PV stenosis | 1 (0.06%) | Anesthetic-induced anaphylactic shock | 1 (0.06%) |
| SVC stenosis & phrenic nerve paralysis | 1 (0.06%) |  |  |

**Supplemental Table 11. Details of complications in all patients**

| **Complication Details (n=8/348 [2.3%])** | | | |
| --- | --- | --- | --- |
| Patient Age | Operation details | Arrhythmia Detail | Complication Detail |
| 78 | Unknown | Reentrant AT of functional RA/ AF of functional LA | TIA, phrenic nerve paralysis |
| 35 | Unknown | Reentrant AT of functional RA | heart failure worsening |
| 35 | Unknown | Reentrant AT of functional RA | heart failure worsening |
| 47 | Unknown | AVNRT of functional RA | transient AV block |
| 37 | Unknown | Reentrant AT of functional RA | heart failure worsening |
| 42 | Unknown | Reentrant AT of functional RA | vascular complication |
| 7 | Patch Closure | AVRT of functional LA | pericardial effusion |
| 39 | Direct Closure | Reentrant AT of functional RA | phrenic nerve paralysis |

**Supplemental Table 12. Details of complications in patients with an ASD closure operation**

| **Complication Details (n=10/216 [2.6%])** | | | |
| --- | --- | --- | --- |
| Patient Age | Operation Details | Arrhythmia Detail | Complication Detail |
| 58 | Extra cardiac conduit | Reentrant AT of functional LA | heart failure worsening |
| 65 | Extra cardiac conduit | Reentrant AT of functional RA | heart failure worsening |
| 32 | APC | Reentrant AT of functional RA | nasal bleeding |
| 43 | LT | Ectopic AT of functional RA | vascular complication |
| 34 | Extra cardiac conduit | AVNRT of functional RA | nasal bleeding |
| 3 | APC | Reentrant AT & ectopic AT of functional RA | vascular complication |
| 4 | APC | Reentrant AT & ectopic AT of functional RA/  Reentrant AT of functional LA | vascular complication |
| 7 | APC | Reentrant AT of functional LA | transient AV block |
| 33 | APC | Reentrant AT & AF of functional RA | sick sinus syndrome |
| 75 | APC | Reentrant AT of functional RA/ Reentrant AT of functional LA | complete AV block |

**Supplemental Table 13. Details of complications in patients with a Fontan operation**

| **Complication Details (n=3/89 [3.4 %])** | | | |
| --- | --- | --- | --- |
| Patient Age | Operation Details | Arrhythmia Detail | Complication Detail |
| 28 | Senning | Reentrant AT of functional RA | cardiac tamponade needed operation |
| 30 | Senning | Reentrant AT &AF of functional RA | hypotension |
| 32 | Senning | Reentrant AT of functional RA | transient AV block |

**Supplemental Table 14. Details of complication in patients with an Atrial Switch operation**

|  | **Bioprosthetic valve** | **Mechanical Valve** | **Complication** |
| --- | --- | --- | --- |
| **Functional Pulmonary Valve (n=90)** | **87 (96.7%)** | **3 (3.3%)** | **1 (1.1%)^*^** |
| **Functional Aortic Valve (n=47)** | **4 (8.5%)** | **43 (91.5%)** | **2 (4.3 %)^†, ††^** |
| **Functional Systemic Atrioventricular Valve (n=40)** | **0 (0%)** | **40 (100%)** | **0 (0%)** |
| **Functional Pulmonary Atrioventricular Valve (n=18)** | **11 (61.1%)** | **7 (38.9%)** | **1 (5.6 %)^†††^** |

**Supplemental Table 15. Incidence of Ablation Complications After Prosthetic Valve Replacement**

***(Bioprosthetic valve)：transient AV block, †(Mechanical valve case): Vascular Complication**

**††(Bioprosthetic valve case): Cardiac tamponade needed drainage, †††(Mechanical valve case): Lead Dislodgement**

| **Complication Details (n=4/195 [2.1 %])** | | | |
| --- | --- | --- | --- |
| Patient Age | Valve position | Arrhythmia Detail | Complication Detail |
| 47 | Functional pulmonary valve  (Bioprosthetic valve) | AVNRT of functional RA | transient AV block |
| 47 | Functional aortic valve  (Mechanical valve) | Reentrant AT & ectopic AT of functional RA | vascular complication |
| 63 | Functional pulmonary atrioventricular valve  (Mechanical valve)  / Functional pulmonary valve  (Bioprosthetic valve) | Reentrant AT of functional RA | lead dislodgement |
| 80 | Functional aortic valve  (Bioprosthetic valve) | Reentrant AT of functional RA  / AF of functional LA | cardiac tamponade needed drainage |

**Supplemental Table 16. Details of complications in patients with valve surgery**

| **Direct Closure**  **(n=19/119 [16.0%])** | **Metal Needle** | **RF Needle** | **Wire** | **No Data** | **Complication** |
| --- | --- | --- | --- | --- | --- |
| **Puncture through prosthetic material**  **(n=0)** | **0 (0%)** | **0 (0%)** | **0 (0%)** | **0 (0%)** | **0 (0%)** |
| **Puncture through native tissue**  **(n=14)** | **1 (7.1%)** | **13 (92.9%)** | **0 (0%)** | **0 (0%)** | **0 (0%)** |
| **Puncture through native tissue**  **/prosthetic material (n=5)** | **0 (0%)** | **4 (80%)** | **0 (0%)** | **1 (20%)** | **0 (0%)** |

**Supplemental Table 17a. Puncture Methods and complication rates in postoperative ASD patients (direct closure)**

| **Patch Closure**  **(n=34/108 [31.5%])** | **Metal Needle** | **RF Needle** | **Wire** | **No Data** | **Complication** |
| --- | --- | --- | --- | --- | --- |
| **Puncture through prosthetic material**  **(n=4)** | **0 (0%)** | **4 (100%)** | **0 (0%)** | **0 (0%)** | **0 (0%)** |
| **Puncture through native tissue**  **(n=26)** | **5 (19.2%)** | **21 (80.8%)** | **0 (0%)** | **0 (0%)** | **1 (3.8%)^*^** |
| **Puncture through native tissue**  **/prosthetic material (n=4)** | **0 (0%)** | **3 (75.0%)** | **0 (0%)** | **1 (25.0%)** | **0 (0%)** |

**Supplemental Table 17b. Puncture methods and complication rates in postoperative ASD patients (patch closure)**

***：pericardial effusion**

| **Catheter Intervention**  **(n=28/38 [73.7%])** | **Metal Needle** | **RF Needle** | **Wire** | **No Data** | **Complication** |
| --- | --- | --- | --- | --- | --- |
| **Puncture through prosthetic material**  **(n=12)** | **11 (91.7%)** | **0 (0%)** | **1 (8.3%)** | **0 (0%)** | **0 (0%)** |
| **Puncture through native tissue**  **(n=16)** | **0 (0%)** | **16 (100%)** | **0 (0%)** | **0 (0%)** | **0 (0%)** |
| **Puncture through native tissue/**  **prosthetic material (n=0)** | **0 (0%)** | **0 (0%)** | **0 (0%)** | **0 (0%)** | **0 (0%)** |

**Supplemental Table 17c. Puncture methods and complication rates in postoperative ASD patients (catheter intervention)**

| **Unknown**  **(n=19/83 [22.9%])** | **Metal Needle** | **RF Needle** | **Wire** | **No Data** | **Complication** |
| --- | --- | --- | --- | --- | --- |
| **Puncture through prosthetic material**  **(n=2)** | **0 (0%)** | **2 (100%)** | **0 (0%)** | **0 (0%)** | **0 (0%)** |
| **Puncture through native tissue**  **(n=14)** | **0 (0%)** | **14 (100%)** | **0 (0%)** | **0 (0%)** | **1 (7.1%)^*^** |
| **Puncture through native tissue/**  **prosthetic material (n=3)** | **0 (0%)** | **3 (100%)** | **0 (0%)** | **0 (0%)** | **0 (0%)** |

**Supplemental Table 17d. Puncture methods and complication rates in postoperative ASD patients (unknown)**

***：TIA & phrenic nerve paralysis**

| **Mustard**  **(n=17/53 [32.1%]])** | **Metal Needle** | **RF Needle** | **Wire** | **No Data** | **Complication** |
| --- | --- | --- | --- | --- | --- |
| **Puncture through prosthetic material**  **(n=13)** | **7 (53.8 %)** | **1 (7.7 %)** | **5 (38.5 %)** | **0 (0%)** | **0 (0%)** |
| **Puncture through native tissue**  **(n=3)** | **0 (0%)** | **1 (33.3%)** | **1 (33.3%)** | **1 (33.3%)** | **0 (0%)** |
| **Puncture through native tissue/**  **prosthetic material (n=1)** | **0 (0%)** | **1 (100%)** | **0 (0%)** | **0 (0%)** | **0 (0%)** |

**Supplemental Table 18a. Puncture methods and complication rates in postoperative atrial switch patients (mustard)**

| **Senning**  **(n=16/36 [32.1%]])** | **Metal Needle** | **RF Needle** | **Wire** | **No Data** | **Complication** |
| --- | --- | --- | --- | --- | --- |
| **Puncture through prosthetic material**  **(n=4)** | **4 (100 %)** | **0 (0%)** | **0 (0%)** | **0 (0%)** | **0 (0%)** |
| **Puncture through native tissue**  **(n=10)** | **4 (60.0 %)** | **6 (60.0%)** | **0 (0%)** | **0 (0%)** | **1 (10.0%)^*^** |
| **Puncture through native tissue/**  **prosthetic material (n=2)** | **0 (0%)** | **2 (100%)** | **0 (0%)** | **0 (0%)** | **0 (0%)** |

**Supplemental Table 18b. Puncture methods and complication rates in postoperative atrial switch patients (Senning)**

***: Cardiac tamponade needed operation**

| **APC Fontan**  **(n=3/85 [3.5%]])** | **Metal Needle** | **RF Needle** | **Wire** | **No Data** | **Complication** |
| --- | --- | --- | --- | --- | --- |
| **Puncture through prosthetic material**  **(n=0)** | **0 (0%)** | **0 (0%)** | **0 (0%)** | **0 (0%)** | **0 (0%)** |
| **Puncture through native tissue**  **(n=3)** | **0 (0%)** | **3 (100%)** | **0 (0%)** | **0 (0%)** | **0 (0%)** |
| **Puncture through native tissue**  **/prosthetic material (n=0)** | **0 (0%)** | **0 (0%)** | **0 (0%)** | **0 (0%)** | **0 (0%)** |

**Supplemental Table 19a. Puncture methods and complication rates in postoperative Fontan patients (APC Fontan)**

| **Extra Cardiac Conduit**  **(n=55/76 [72.4%])** | **Metal Needle** | **RF Needle** | **Wire** | **No Data** | **Complication** |
| --- | --- | --- | --- | --- | --- |
| **Puncture through prosthetic material**  **(n=48)** | **30 (62.5%)** | **3 (6.3%)** | **15 (31.3%)** | **0 (0%)** | **3 (6.3%)^*,†, ††^** |
| **Puncture through native tissue**  **(n=6)** | **0 (0%)** | **5 (83.3%)** | **0 (0%)** | **1 (16.7%)** | **0 (0%)** |
| **Puncture through native tissue/**  **prosthetic material (n=1)** | **0 (0%)** | **1 (100%)** | **0 (0%)** | **0 (0%)** | **0 (0%)** |

**Supplemental Table 19b. Puncture methods and complication rates in postoperative Fontan patients (extracardiac conduit)**

***:nasal bleeding、†：Heart Failure Worsening、††：Heart Failure Worsening**

| **Intra Atrial Graft**  **(n=8/15 [53.3%])** | **Metal Needle** | **RF Needle** | **Wire** | **No Data** | **Complication** |
| --- | --- | --- | --- | --- | --- |
| **Puncture through prosthetic material**  **(n=3)** | **2 (66.7%)** | **1 (33.3%)** | **0 (0%)** | **0 (0%)** | **0 (0%)** |
| **Puncture through native tissue**  **(n=4)** | **0 (0%)** | **3 (75.0%)** | **1 (25.0%)** | **0 (0%)** | **0 (0%)** |
| **Puncture through native tissue/**  **prosthetic material (n=1)** | **1 (100%)** | **0 (0%)** | **0 (0%)** | **0 (0%)** | **0 (0%)** |

**Supplemental Table 19c. Puncture methods and complication rates in postoperative Fontan patients (intraatrial graft)**

| **LT**  **(n=17/36 [47.2%])** | **Metal Needle** | **RF Needle** | **Wire** | **No Data** | **Complication** |
| --- | --- | --- | --- | --- | --- |
| **Puncture through prosthetic material**  **(n=7)** | **6 (85.7%)** | **1 (14.3%)** | **0 (0%)** | **0 (0%)** | **0 (0%)** |
| **Puncture through native tissue**  **(n=5)** | **0 (0%)** | **5 (100%)** | **0 (0%)** | **0 (0%)** | **0 (0%)** |
| **Puncture through native tissue/**  **prosthetic material (n=5)** | **2 (40.0%)** | **3 (60.0%)** | **0 (0%)** | **0 (0%)** | **0 (0%)** |

**Supplemental Table 19d. Puncture methods and complication rates in postoperative Fontan patients (lateral tunnel)**
